# Feasibility and Acceptability of a Task-Shifted Intervention to Enhance Adherence to HIV Medication and Improve Depression in People Living with HIV in Zimbabwe, a Low Income Country in Sub-Saharan Africa

**DOI:** 10.1007/s10461-016-1659-4

**Published:** 2017-01-06

**Authors:** Melanie Abas, Primrose Nyamayaro, Tarisai Bere, Emily Saruchera, Nomvuyo Mothobi, Victoria Simms, Walter Mangezi, Kirsty Macpherson, Natasha Croome, Jessica Magidson, Azure Makadzange, Steven Safren, Dixon Chibanda, Conall O’Cleirigh

**Affiliations:** 1 Health Service & Population Research Department, Centre for Public Mental Health, Institute of Psychiatry, Psychology & Neuroscience, King’s College London, Box PO 60, De Crespigny Park, London, SE5 8AF UK; 2 College of Health Sciences, University of Zimbabwe, Harare, Zimbabwe; 3 Zimbabwe AIDS Prevention Project, Department of Community Medicine, University of Zimbabwe, Harare, Zimbabwe; 4 Parirenyatwa General Hospital, Harare, Zimbabwe; 5 Department of Infectious Disease Epidemiology, London School of Hygiene & Tropical Medicine, London, UK; 6 Massachusetts General Hospital/Harvard Medical School, Boston, MA USA; 7 Department of Psychology, University of Miami, Miami, FL USA

**Keywords:** Adherence, Depression, Sub-Saharan Africa, Problem solving, Intervention

## Abstract

Using a pilot trial design in an HIV care clinic in Zimbabwe, we randomised 32 adults with poor adherence to antiretroviral therapy and at least mild depression to either six sessions of Problem-Solving Therapy for adherence and depression (PST-AD) delivered by an adherence counsellor, or to Enhanced Usual Care (Control). Acceptability of PST-AD was high, as indicated by frequency of session attendance and through qualitative analyses of exit interviews. Fidelity was >80% for the first two sessions of PST-AD but fidelity to the adherence component of PST-AD dropped by session 4. Contamination occurred, in that seven patients in the control arm received one or two PST-AD sessions before follow-up assessment. Routine health records proved unreliable for measuring HIV viral load at follow-up. Barriers to measuring adherence electronically included device failure and participant perception of being helped by the research device. The study was not powered to detect clinical differences, however, promising change at 6-months follow-up was seen in electronic adherence, viral load suppression (PST-AD arm 9/12 suppressed; control arm 4/8 suppressed) and depression (Patient Health Questionnaire—4.7 points in PST-AD arm vs. control, adjusted p value = 0.01). Results inform and justify a future randomised controlled trial of task-shifted PST-AD.

## Introduction

HIV remains one of the biggest challenges facing sub-Saharan Africa (SSA), with 25.5 million people estimated to be infected in 2015, 1.4 million new infections, and 72% of global HIV-related deaths [1]. In Zimbabwe, there are 1.4 million people living with HIV (PLWH), which is 14.7% of the adult population [2]. Among PLWH aged 15–64 who know their status, 86.4% self-report current use of antiretroviral therapy [3]. For those who have tested HIV-positive, the global target for 2020 is to have 90% on ART and for 90% of those to be virally suppressed [4]. It is estimated that ART adherence levels of 80–95% are required in order for individuals to achieve viral suppression [5].

As confirmed through systematic reviews [6], psychological interventions that emphasise motivational and problem-solving approaches have in some settings been shown to enhance ART adherence and/or viral suppression in treatment-experienced patients [7, 8]. However, such approaches are rarely available in low-income countries, due in part to the lack of behavioural medicine specialists. There is thus much merit in research on psychological and behavioural interventions, which could potentially be delivered by non-specialists through task-sharing. We have described elsewhere qualitative research to learn about barriers to adherence in Zimbabwe [9]. We have also described use of those data to inform adaptation of Life-Steps [10], a structured adherence intervention with educational, motivational and problem-solving components. Modifications were made in terms of language, session length, tailoring of content for delivery by lay counsellors, and inclusion of culturally competent probes [11]. We called the culturally adapted psychological intervention for adherence *Nzira Itsva*, which translates as New Direction.

In SSA, as is the case world-wide, depression is one of the key predictors of poor adherence to ART [12]. Even in mild form, depression is associated with a 42% lower likelihood of achieving good adherence to ART compared to those without depressive symptoms (pooled OR = 0.58, 95% CI 0.55–0.62) [13]. This is important for two reasons. First, significant depression symptoms are found in up to half of all people living with HIV [14]. Second, the characteristic symptoms and impairments found in depression are likely to interfere with critical functions necessary to maintain good adherence, such as information processing, planning, problem-solving, and activation of adherence behaviours [15, 16]. Classical symptoms and impairments found in depression include repetitive negative thinking, poor attention, persistently low mood, slowed cognition, and diminished executive functioning [17–19]. Poor adherence to ART is likely to be the mechanism by which depressive symptoms predict higher rates of progression to AIDS and worse survival for PLWH [20], although depression may also have a direct effect on immune status [21].

Despite the strong link between depression and poor adherence, and evidence that psychological and pharmacological treatments improve depressive symptoms and general function for PLWH with depression, it is striking that treatment for depression is mostly absent in HIV facilities in low-income settings. A key reason for this is the lack of mental health specialists and of culturally-adapted therapies. In SSA, the median number of mental health professionals (1.7 per 100,000 population [22]) is less than one fiftieth the rate in the United States (92.7 mental health professionals per 100,000 population [23]). Zimbabwe, with a 13 million population, has only two clinical psychologists and 10 psychiatrists in government service. As is common in low-income countries, antidepressant drugs are also in limited supply, and most staff in general health care settings are not trained to prescribe them.

In four trials in the US, Cognitive-Behavioural Therapy (CBT) for depression was integrated with a multi-component cognitive-behavioural adherence intervention. Benefits were seen for adherence and/or viral suppression as well as for depression [24–27]. There is only weak evidence that treatment of depression alone without additional adherence counselling improves adherence to ART [28]. Also a large randomised controlled trial of antidepressant therapy found no significant effect on adherence, despite good benefit for depression [29]. To date, there have been no randomised trials of psychological interventions for depression and adherence in low income countries. Our intervention combines Problem-Solving Therapy (PST) for depression, locally known as ‘Opening Up the Mind’ or *Kuvhura Pfungwa* [30, 31], with *Nzira Itsva* for adherence. In the local setting we named the integrated treatment for depression and adherence ‘TENDAI’ (meaning ‘thankful’ in the Shona language). Problem-Solving Therapy is a brief evidence-based intervention derived from social problem-solving, in which patients are taught a structured approach to identify problems and find workable solutions [32]. It is an attractive option for low-resource settings, because unlike CBT, it does not require extensive training or complex skills [33]. We have further embedded PST in a stepped care model, both to maximize efficiency of resource allocation [34] and to incorporate principles to guide treatment choices [35].

The aims of this feasibility study were to measure acceptability of the PST-AD intervention for participants and for clinic staff; test methods that would inform a future randomised trial; and gather data to inform a sample size calculation for a future trial.

## Methods

### Setting

We recruited participants over 14 weeks (September–November 2014, January 2015) at the Parirenyatwa Hospital Family Care Centre (PHFCC), a government clinic affiliated with the University of Zimbabwe College of Health Sciences (UZCHS). This clinic provides comprehensive HIV care for around 3000 adults and 1000 children on ART. Patients attend an average of 12 appointments annually. Registered patients receive clinical reviews, ART, HIV-related opportunistic infection prophylaxis, CD4 T cell profile and annual viral load tests at no cost, but are required to pay for additional laboratory and diagnostic evaluations. Patient clinical data is entered on an electronic medical record as well as hand written into a notebook that is retained by the patient and serves as their personal, mobile health record. Medical doctors provide the bulk of the primary care, with nurses having limited prescribing capability. Patients suspected of having a mental disorder could be referred to the psychiatry clinic in a separate building, although in practice only about six referrals were being made per month with no records of the outcome.

### Study Design

The design was an individualised randomised controlled trial in which an experimental intervention was compared to enhanced usual care (EUC). All participants in the experimental arm had experienced usual care when they initiated ART, and all counsellors trained in the experimental treatment had previously used usual care. Therefore, both patients and counsellors had previous experience of usual care to compare with.

Study outcomes are summarised in Table 1.

**Table 1:** Study Outcomes

Aim	Outcome
Feasibility of methods for conducting a trial in this setting	Recruitment: Proportion of screened participants eligible to take part, proportion of eligible participants consenting, time taken to recruit Trial retention: Proportion of participants able to be followed up at 4 months Feasibility of using routine clinic data: Monitor missed appointments, CD4 and viral load, as measured by proportion of data gathered. Feasibility of using the Wisepill device to measure electronic adherence: Number of measurements recorded; participants’ experiences of using the device
Acceptability of the intervention for participants and clinic staff	Proportion of sessions attended, proportion of sessions where non-specialist collected depression measure Number of weeks to complete six sessions Exit interviews with participants and staff
Feasibility of collecting clinical outcome measures at baseline and at follow-up 6 months after last intervention session	Depression symptom score: Using the Patient Health Questionnaire for depression and the Shona Symptom Questionnaire for common mental disorders [36, 37, 59] Electronic ART adherence score: Measured by the Wisepill device for 14 days at baseline and follow up. The score is the mean percentage of pills taken on time (±1 h) over the past 14 days. We defined good adherence as ≥90% in the past 14 days [60] Self-report adherence: Based on shortened version of the AIDS Clinical Trials Group questionnaire [40] to measure any missed ART doses in last 7 days, last 30 days, or last 3 months Proportion of participants with one or more missed HIV care appointments in the last 3 months: Derived from the patient record system Viral load: Detectable viral load >200 copies/ml; mean viral load, derived from the patient record system

#### Recruitment

Recruitment was conducted through an announcement by the trained research assistant (RA) asking for volunteers while patients waited for their clinical evaluations or laboratory tests, and through medical staff directly referring patients who were considered at risk of poor adherence. If a patient was interested, they were screened by the RA in a confidential space. The following inclusion criteria were used: aged 18 or over; on ART for at least 4 months; and risk of poor adherence as indicated by any one of the following: (i) missed at least one clinic appointment in the last 3 months; (ii) falling CD4 count or detectable viral load in the previous 6 months; or (iii) self-report of poor adherence (admitting to having missed one or more doses, taking treatment late, or being forgetful with treatment). Those meeting criteria were interviewed and to be included had to (iv) screen positive for at least mild depression, as defined by scoring at least 5 on the Patient Health Questionnaire, and/or at least 9 on the Shona Symptom Questionnaire.

#### Depression Measures

We screened for depression using the Patient Health Questionnaire (PHQ-9). The PHQ-9 asks about symptoms over the past 2 weeks, deriving its scoring system from the DSM-IV criteria for depressive disorders. Each of the nine items is scored from 0 (not at all) to 3 (nearly every day). It is used as a continuous score ranging from 0 (no depressive symptoms) to 27 (all symptoms occurring daily); as a binary measure, with a cut-point of 10 or greater recommended in the US, (sensitivity and specificity for major depression of 88%), and as bands, with scores of 5–9, 10–14, 15–19, and 20 plus representing mild, moderate, moderately severe, and severe depression, respectively [36]. As a binary measure, the optimal cut-off for PHQ-9 in Zimbabwe is ≥11, which provides a sensitivity of 85% (95% CI 78–90%) and specificity of 69% (95% CI 59–77%) against a SCID diagnosis of depression (Cronbach’s α = 0.86) [37]. We also used the Shona Symptom Questionnaire (SSQ-14). The SSQ-14 was developed in Zimbabwe, and is an easy to use, binary measure to screen for depression as well as other common mental disorders, e.g. anxiety. With the optimal cut-off of ≥9, the sensitivity and specificity for the SSQ-14 against a diagnosis of either depression and/or generalised anxiety is 84% (95% CI 78–89%) and 73% (95% CI 63–81%) respectively. Internal reliability is high (Cronbach α = 0.74) [37].

At 6-months follow-up, we used both the PHQ-9 and the SSQ-14. We were interested to use both scales in the feasibility study. The PHQ-9 is known to be sensitive to change, serves as a screen for depression, and is validated locally. The SSQ-14 was developed in Zimbabwe, includes indigenous idioms, and is user-friendly, being a simple binary questionnaire. It has been validated for depression and other common mental disorders. If the SSQ-14 does prove to be  sensitive to change, it could be a useful tool for a future trial given its user-friendliness.

#### Exclusion Criteria

Patients were excluded if they scored ≤6 on the International HIV-Dementia Scale [38]; indicated suicidal intent on the P4 Suicidality Screener [39]; or were assessed by interviewer to be too unwell, intoxicated, or distressed to take part. All those meeting study inclusion criteria were invited to take part.

#### Randomisation

**Fig. 1:**
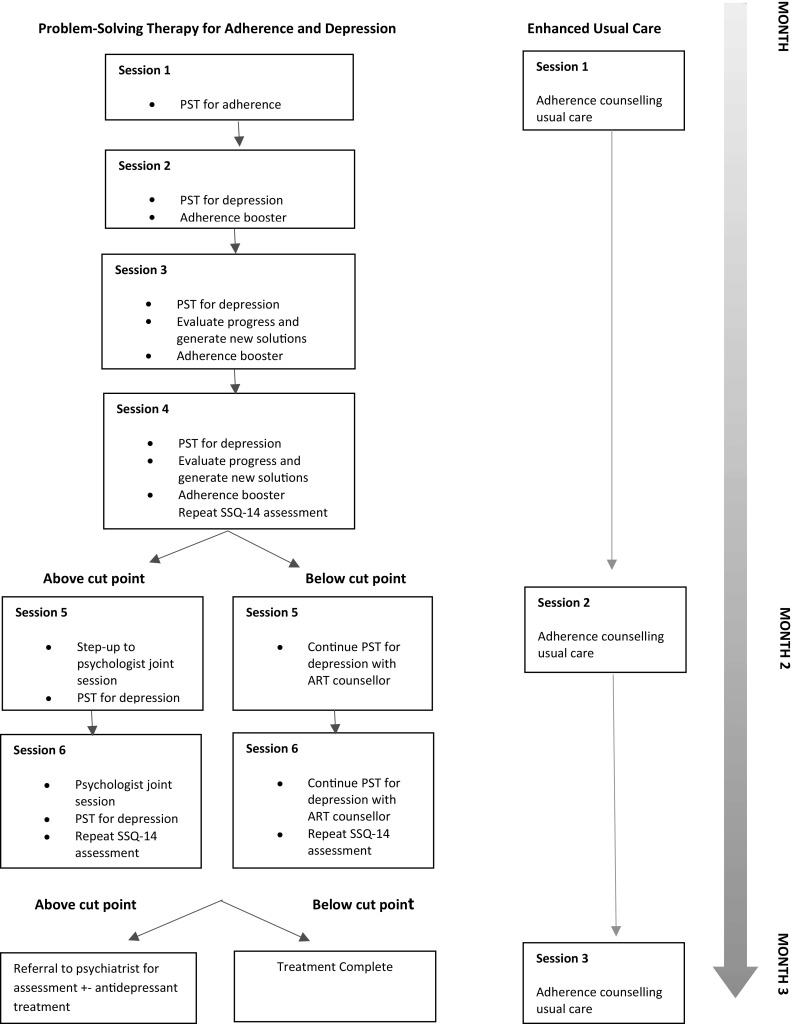
Flow-chart showing the experimental and enhanced usual care interventions

After giving written informed consent, patients were randomised in a 1:1 ratio to intervention or EUC arms. Randomisation was conducted by participants selecting one numbered card at random from a bag; each number had been pre-allocated to either the intervention or EUC arm. We aimed to recruit 40 participants over 4 months. The flow of recruitment and randomisation is shown in Fig. 1.

#### Baseline Assessment

For individuals who met all study criteria and provided informed consent, the trained RA conducted the baseline assessment on the day of recruitment. Data collected from the participant included socio-demographics, ART regimen and duration of treatment, AUDIT-C screening for alcohol misuse, and self-reported adherence [40]. Data collected from the participants’ clinical notebook and/or electronic records included the two previous viral load and CD4 test results and any missed HIV care appointments in last 3 months.

#### Electronic Medication Event Monitoring

Electronic pill bottle monitoring is proven to be a valid and reliable measure of adherence [41] and can be carried out with tools such as Wisepill. Wisepill is a portable medication dispenser that has a cell phone communication chip which sends a signal (medication event) each time the dispenser is opened. Participants in both arms were trained to use a Wisepill device and were asked to store and take their medication from it for 2 weeks prior to their first therapy session. Adherence records were available to the clinicians and researcher through a Wisepill database.

#### Blinding

Participants were at least partly blinded to their group allocation as both arms were offered extra sessions of counselling, which is different from standard care. We piloted independent assessment of follow-up data on self-report outcomes of depression and adherence in 25% of participants, but resources precluded doing this for all participants. Data on missed appointments, viral load, and Wisepill adherence at follow-up were collected and analysed by researchers blinded to study arm.

We collected qualitative data by interviewing a randomly selected sub-sample of 25% of participants (7/28). Qualitative data were analysed by two researchers outside the core research team who were not aware of the content of sessions in the intervention or EUC arms. The two researchers were trained in qualitative data analysis and had a background in mental health in low-income countries (see acknowledgements).

#### Fidelity to the Intervention

The supervising psychologist and a research psychologist independently rated fidelity in a random 15% of recordings (24 sessions) using a therapist checklist adapted from one used for similar interventions [24]. Six evaluations were conducted each of a session 1, 2, 3 and 4 of the intervention. Sessions were rated on the presence or absence of key competencies for each session, for instance setting an agenda, reviewing adherence in the past week, or reviewing progress on the solution generated in the previous session. Competencies covered both the depression and the adherence component of the intervention.

Fidelity ratings were carried out 3 months after the study had ended. Disagreements were resolved by discussion and consensus through a 3-way face-to-face discussion with the first author.

#### Follow-Up

Participants were followed up at 6 months on both measures of depression and adherence, using the same measurement tools as at recruitment. Follow-up took place in the respective clinic, however if the patient was unavailable or did not arrive for follow-up, they were contacted and assessed by telephone.

### Experimental Intervention

#### Problem-Solving Therapy for Depression and HIV Medication Adherence (PST-AD)

The intervention, locally named the TENDAI intervention, is delivered over 6 weekly sessions: the first two sessions 50 min; sessions 3–6 30 min. Session 1 is *Nzira Itsva*, the culturally and linguistically adapted Life-Steps adherence intervention [10, 42]. As described elsewhere [11], *Nzira Itsva* has a series of motivational, informational, problem-solving and behavioural steps. In the local context, we had found that clients commonly responded by wanting to work on generating solutions to barriers to getting to appointments (such as very constrained finances or lack of autonomy at work or home to attend the clinic), asking questions of the medical team, having a regular pill-taking schedule (which was especially hard where the person had not disclosed their status in the household) and, for women, dealing with ways in which their husband might be interfering with pill-taking. This first session is operationalized with detailed daily adherence data over the past 2 weeks generated by the Wisepill electronic device.

Session 2–6 follow classical PST for depression including psycho-education about depression; eliciting, listing and reflecting back to the participant their problems; helping them to select one problem from the list to focus on; brainstorming solutions; rating them according to importance and feasibility; choosing a solution; and making a plan to implement it over the next week [43]. Subsequent sessions include evaluating progress and may include generating new solutions and tackling different problems. The counsellors were trained to include 10–15 min on adherence barriers in session 2–6, building on the first session, checking progress, and using the PST approach to try to find solutions for additional barriers. The counsellor met weekly with the psychologist to discuss her case-load.

#### Stepped Care

At session 4, the counsellor repeated the SSQ-14 screening scale for depression. If the SSQ-14 score remained above 8/14 and/or the patient was having difficulty generating solutions to problems, the ADC had the option to step up the intervention. This involved asking the psychologist to see the client in session 5 as a joint session. The psychologist could advise the counsellor and/or deliver Session 6 also as a joint session.

The ADC repeated the SSQ-14 assessment at Session 6 for those she suspected were not improved. Those scoring above the cut-off point could be discussed again with the psychologist or referred to a psychiatrist for an assessment for antidepressant treatment (Step 3 of the intervention). Although we had considered using antidepressants, such as Fluoxetine, for Step 2 care, we found they were not available in the general healthcare pharmacy in the HIV clinic, and were poorly available more generally in Zimbabwe. Also, the medical staff in the HIV clinic were neither willing nor confident to prescribe these drugs. There were additional concerns about pill burden, possible interactions with antiretroviral therapy, and lack of desirability of using Tricyclic Antidepressants, which would be the most available option. We thus focused on psychological therapy for Step 2. Although the emphasis remained on problem-solving therapy, at Step 2 it was provided by someone more qualified than the adherence counsellor.

One Adherence Counsellor (ADC) trained in PST-AD delivered the intervention for all the participants in the PST-AD arm. Although we had trained three others in PST-AD, one of these emigrated, and two ADCs were moved to different departments within the clinic, as they were perceived by the matron to have new skills which could be deployed in critical clinical areas.

### Control Intervention

#### Usual Care Counselling for Adherence to ART

Counselling is provided by ADCs. This cadre are usually primary care counsellors or nurse aides who have secondary school education and 6 months of training in HIV/AIDS basic counselling. Counselling sessions include information about HIV/AIDS, safe sex practices, diet, keeping a positive attitude towards living with HIV, and why ART is important. Advice is given on how to take medication and any social problems that emerge during the discussion. Counselling is routinely provided under four conditions: (1) when a patient initiates ART; (2) when a patient has ‘defaulted’ from their treatment i.e. did not collect medication for some months, or (3) when a patient is clinically suspected of poor adherence through having a falling CD4 count or high viral load, or (4) admits to poor adherence through self-reported difficulty in compliance. Those initiating ART or those who ‘default’ treatment attend one group-based and one individual session of ART counselling. Those considered at risk of poor adherence are referred for one individual session, although depending on need, as perceived by the counsellor or clinician, that could in rare cases be repeated.

#### Enhanced Usual Care (EUC)

Four ADCs who had not been trained in the PST-AD intervention delivered the EUC. We enhanced usual care by (1) increasing usual care from one session only, to one session a month for 3 months, and (2) ensuring continuity of the same counsellor for all sessions. ADCs providing EUC were asked to repeat the information they normally provide. They were not trained in the local version of Life-Steps.

### Data Analysis

Study data were collected and managed using REDCap electronic data capture tools hosted at the University of Zimbabwe, College of Health Sciences [44]. All analysis was undertaken using Stata (V.14). Binary outcomes were analysed using Fisher’s exact test due to small numbers. To analyse the continuous depression and adherence outcomes by arm, linear regression models were used, adjusting for baseline outcome score. An intention to treat analysis was then performed to include every subject who was randomized according to randomized treatment assignment. Intention to treat analysis reflects the real-world clinical scenario because it admits noncompliance and protocol deviations [45]. It thus minimizes type I error due to a cautious approach and allows for the greatest generalizability [46].

Semi-structured interviews were coded and analysed in NVivo10 using grounded theory principles [47] and thematic content analysis [48]. For each interview, we created a list of conceptual components (‘opening coding’) and re-categorised these ideas into initial themes based upon the study objectives of investigating the acceptability and potential benefits of the PST-AD intervention [49]. Initial themes were adapted into a framework through a process of iteration, and additional themes were added as determined by the data.

### Ethics

The study was approved by the Medical Research Council of Zimbabwe and the Research Council of Zimbabwe (MRCZ/A/1736), the Joint Parirenyatwa Hospital and College of Health Sciences Research Ethics Committee (JREC 18/13), and King’s College London College Research Ethics Committee (PNM/13/14-158). The trial was also registered with the Pan African Clinical Trials Registry (PACTR201511001150307).

All participants provided informed consent. Participants were compensated with USD3 per visit for public transport costs and were provided with refreshments.

## Results

### Recruitment

**Fig. 2:**
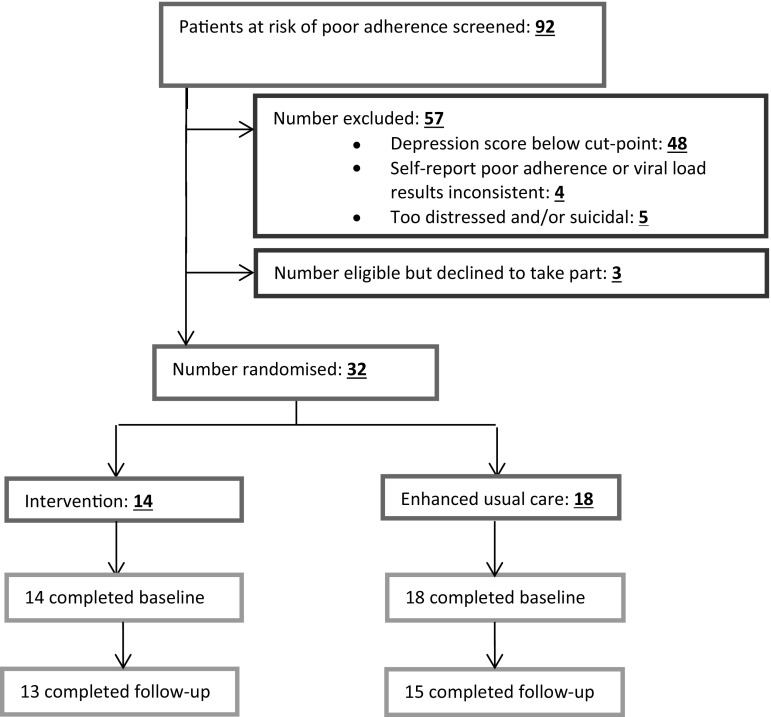
Flow-chart showing the progress of participants from screening to follow-up

Over 14 weeks, clinic doctors referred 94 patients with a detectable viral load, of whom 79/94 (84%) were willing to be screened. We screened a further 13 who volunteered among those waiting in the blood test queues as having a high viral load and/or having problems with adherence. As shown in Fig. 2, 44/99 (47.8%) scored at or above the cut-point for depression on the PHQ-9 and/or on the SSQ-14, of whom four were excluded as they later proved not to have any of the clinical criteria for poor adherence to ART. Five patients were excluded due to being severely distressed and/or suicidal. Only three declined to take part, two citing distance from the clinic as a barrier to attending counselling sessions and one saying he was busy with exams. As shown in Fig. 2, 14 patients were randomised to the intervention arm and 18 to the control condition, and 28 completed follow-up. As shown in Table 2, those in the intervention arm did not differ in terms of gender (65% female) or marital status, but were less likely to be employed or to have tertiary education than those in the control arm.

**Table 2:** Characteristics of participants at baseline

	Intervention arm (N = 14)		Enhanced usual care arm (N = 18)		Total (N = 32)	
	N (%)	Median (IQR)	N (%)	Median (IQR)	N (%)	Median (IQR)
Age (years)		41.8 (33.7–45.3)		35.8 (24.1–40.6)		37.8 (24.6–44.9)
Gender: female	9 (64.3)		12 (66.7)		21 (65.6)	
*Marital status*						
Married	7 (50.0)		9 (50.0)		16 (50.0)	
Single	5 (35.7)		7 (38.9)		12 (37.5)	
Widowed	2 (14.3)		2 (11.1)		4 (12.5)	
*Highest education*						
Pre-primary and primary school	4 (28.6)		5 (27.7)		9 (28.1)	
Secondary and high school	9 (64.3)		9 (50.0)		18 (56.3)	
Tertiary	1 (7.1)		4 (22.2)		5 (15.6)	
*Employment status*						
Not employed	8 (57.1)		5 (27.8)		13 (40.6)	
Employed (full-time, part-time and self-employed)	6 (42.9)		12 (66.6)		18 (56.3)	
Student	0		1 (5.6)		1 (3.1)	
Food insecurity: skipped meals in the last month	3 (21.4)		3 (16.7)		6 (18.8)	
Time on ART (years)		5.5 (2.3–7.6)		3.9 (3.0–6.4)		4.3 (2.9–6.5)
*ART regimen*						
First line	12 (85.7)		14 (77.8)		26 (81.3)	
Second line	2 (14.3)		4 (22.2)		6 (18.7)	
Viral load non-suppressed (>200 copies/ml)	13 (92.9)		17 (94.4)		30 (93.8)	
CD4 count (cells/mm^3^)		300 (144–403)		325 (140–561)		316 (142–438)
AUDIT-C alcohol screen	0		2 (11.1)		2 (6.3)	

### Attendance and Session Completion

For those randomised to the 6-session intervention, the mean number of sessions attended was 5.2 (SD 1.6). Ten of the 14 participants (71%) completed all six sessions, 2 (14%) completed five sessions, and two (14%) completed one or two sessions. For those who completed six sessions, the median duration from first to last session was 8 weeks. For those randomised to the 3-session EUC, the mean number of sessions was 2.4 (SD = 0.6). For 20% of participants, the research assistant made more than one phone call to re-book their next session.

### Follow-Up at 6 Months

Trial retention was 88% (28/32,) with 13 intervention participants (93%) and 15 control participants (83%) followed up. Two follow-up interviews were conducted by telephone. The four participants lost to follow-up did not appear to differ significantly from the remaining 28 patients in their baseline characteristics of gender, age or marital status.

### Fidelity to the Intervention

Fidelity to sessions 1 and 2 was high (83 and 88% respectively) out of 10 competencies each. For sessions 3 and 4, fidelity dropped to 63 and 53% respectively. This was almost entirely due to the drop in fidelity to the adherence component of sessions, which was only 33%. The main gaps were that the counsellor commonly omitted to review adherence in the past week, or to review additional adherence barriers or work with the client to develop ways to overcome them. For sessions 3 and 4, fidelity was 75% for the competencies for PST for depression.

### Feasibility of Measuring Clinical Outcomes (Table 3)

#### Depression Scale Scores

We were able to collect data on depression for all participants at baseline and for 28/32 (87.5%) at follow-up (two participants provided follow-up interviews by phone). The Adherence Counsellor was able to measure depression at session 4 for 100% of participants in the PST-AD arm. Being in the intervention arm compared to the EUC arm was associated with 4.7 points lower PHQ-9 score at follow-up (95% CI −8.2, −1.3 p = 0.010) controlling for baseline PHQ-9 score (N = 28, 25 residual degrees of freedom, F = 3.95, adjusted R^2^ = 18%) (Table 3). The participants in the intervention arm also had 1.13 points lower mean SSQ-14 score at follow-up (95% CI −3.25, 0.99) as compared to the EUC arm, after adjusting for the effect of baseline SSQ-14 score, but the effect was not significant (p = 0.284, N = 28, 25 residual degrees of freedom, F = 5.55, adjusted R^2^ = 25%). In terms of clinical significance, in the intervention arm, the average depression score on the PHQ-9 fell from ‘moderate depression’ range to ‘no depression’ range. In the EUC arm, average depression score on the PHQ-9 also fell, but remained in the ‘mild depression’ range at follow-up. Only one of the 13 intervention participants (8%) but 6 of the 15 EUC participants (40%) were still above the cut-off for depression on either the PHQ-9 or the SSQ-14.

**Table 3:** Clinical Outcomes

	Intervention arm		EUC arm	
	Baseline (n = 14)	Follow-up (n = 14)	Baseline (n = 18)	Follow-up (n = 18)
Viral suppression	1 (7.1%)	9 (75%)^b^	1 (5.6%)	4 (50%)^c^
Switched to 2nd or 3rd line regimen		6 (42.9%)		7 (38.9%)
Missed at least one HIV care appointment in last 3 months: N (%)	4 (28.6%)	3 (21.4%)	4 (22.2%)	8 (44.4%)
Number of missed HIV care appointment in last 3 months: mean (SD)	0.36 (0.63)	0.29 (0.61)	0.39 (0.78)	0.72 (0.96)

	Baseline (n = 14)	Follow-up (n = 13)	Baseline (n = 18)	Follow-up (n = 15)
Depression Shona Symptom Questionnaire Score: mean (SD)	8.5 (1.9)	3.8 (3.3)	9.3 (1.2)	5.5 (2.7)
Patient Health Questionnaire score: mean(SD)	13.8 (2.2)	3.1 (3.1)	9.2 (3.3)	5.7 (3.9)
Self-report missed dose(s) within last month: N (%)	3 (21.4%)	1 (7.7%)	4 (22.2%)	1 (6.7%)
Electronically-measured adherence ≥90%: N (%)	8 (57.1%)	9 (81.8%)^b^	7 (43.8%)^b^	8 (57.1%)^a^
Electronically-measured adherence: mean (SD)	85.3 (20.8)	93.8 (10.2)^b^	71.4 (30.6)^b^	85.3 (26.2)^a^

^a^missing data on 1 participant

^b^missing data on 2 participants

^c^missing data on 10 participants

#### Missed HIV Care Appointments

It proved feasible to measure missed appointments through the clinic record system. At baseline a similar proportion in the EUC arm (22%) and in the PST arm (29%) had missed appointments in the previous 3 months. However, at follow up, a greater proportion in the EUC arm (44%) than in the PST-AD arm (21%) had missed appointments in the prior 3 months. This was not statistically significant (p = 0.266).

#### Viral Load and CD4

We found considerable limitations in feasibility of using the clinic record system to determine HIV viral load quantification and CD4 T cell counts at baseline and follow-up. For viral load, this was due to (i) the long period between having blood taken and result being available on the record system (mean 101 days delay at baseline and 130 days delay at follow-up), (ii) errors in dates (e.g. mixing up date of blood taken with date result entered), (iii) timing of blood tests (for instance, within 1 month of the intervention which would be too soon to expect a change in viral suppression), (iv) non-random testing i.e. those with a lower previous viral load were less likely to have a follow-up test. Viral load data were missing for 12/32 participants (38%). At baseline, one intervention arm participant and one in the EUC arm were virally suppressed. At follow up 9/12 (75%) in the PST-AD arm compared to 4/8 (50%) in the EUC arm achieved viral suppression, but the result was not statistically significant (p = 0.356). On CD4 count outcomes, most participants lacked data due to the rarity of follow-up CD4 being requested.

#### Self-Report Adherence

The proportion of participants who self-reported missing a dose in the past 4 weeks decreased for both arms. In the intervention arm this fell from 21 to 8% and in the EUC arm from 22 to 7% (Table 3). There was no difference between arms at follow-up (p = 0.722).

#### Electronically-Measured Adherence

We collected an adherence score for 30/32 participants (94%) at baseline and 25/28 (89%) at follow-up. Missing data was due to low battery in the device (needing charging), network problems, or expiry of cell phone credit. In one case the participant said the Wisepill had been stolen. As shown in Table 3, the percentage of participants with good adherence (≥90% of pills taken on time) increased for both groups although there was no statistically significant difference between arms at follow-up (p = 0.190). In the intervention arm there was a greater absolute increase in good adherence (25%) than in the EUC arm (14%). Mean adherence also increased for both arms. At an individual level, mean change in the intervention arm was 11.2 (95% CI 0.6, 21.9) and in the EUC arm was 11.6 (95% CI −2.1, 25.3). A linear regression model found no association between arm and adherence score at follow-up, controlling for baseline adherence score (coefficient = 2.6, 95% CI −11.2, 16.5, p = 0.695, N = 24, 21 residual degrees of freedom, F = 7.76, adjusted R^2^ = 37%).

#### Stepped Care in the Intervention Arm

Thirty-six percent (5/14) of participants were ‘stepped up’ to be seen by the psychologist at session 4. During exit interviews, the ADC said in most of the cases, by session 3, she had been aware that she was going to need to refer the participant to the psychologist, because she was struggling to assist the participant to generate solutions. Two of these 14 (14%) were then further referred to Step 3 for an antidepressant.

#### Experience of Participating in a Pilot Randomised Controlled Trial

We conducted 35 exit interviews, 27 with participants (12 PST-AD arm, 15 EUC arm) and six with ADCs (two trained in PST-AD and four trained in EUC but not in PST-AD). Key themes that emerged were the acceptability and perceived benefits of the PST-AD intervention, the sense of being “helped” by the electronic medication event monitoring, and the contamination that occurred in the EUC arm.

#### Acceptability of the Intervention (See Table 4)

Interviews with intervention arm participants notably revealed high levels of acceptability and perceived benefits of the intervention with many participants saying that the intervention was “great” and that the intervention “should help everyone because this is extremely beneficial”. Participants noted that it had “educated” them about adherence and also about depression. Participants could now identify why adherence is necessary: “If I do not adhere it means that the viral load will rise and in turn will affect my health”, understand terminology commonly used by health care professionals: “I benefitted a lot from all the sessions—I did not understand the difference between viral load and CD4 but through the sessions now I know the difference”, and start incorporating reminders into their daily routine: “I now make use of reminders for myself, such as stickers and alarms so that I will be able to take my pills on time”. Participants stated the intervention “should be continued” in particular it “should consider educating more people on depression and adherence”. Ninety-one percent (11/12) of intervention arm participants felt that the counselling sessions had influenced the way in which they take their medication, with 75% (9/12) stating that they were now able to take their pills on time. This compared to 47% (7/15) of the EUC participants. The animation helped the clients to see why taking medication on time was so important, motivating them to problem-solve around their barriers to adherence so they could follow what the counsellors and the doctors asked them to do. Many participants in the intervention arm and some in the EUC arm said that the impact of the intervention included learning how to, for example, problem-solve, manage stress, generate forms of employment, deal with familial problems and lead a healthier lifestyle. Some intervention arm participants said that through talking about their financial difficulties with the counsellor, they have “started their own business now”, “found employment” or “begun a project”. One participant noted, “I have learnt that I can do something with the little I have and become someone. In my case I am able to translate English into French and Lingala, I am translating for other people especially in church and I am saving out of it”. The guidance and support of the ADCs helped participants develop ways to manage their financial difficulties: “With the help of the counsellor, I managed to come up with a solution to my financial problem. I have now started my own business of selling food.” Perceptions amongst participants appear to be shared by the counsellors with experience of the PST-AD intervention. ADCs highlighted how the intervention “improves adherence”, “helps clients understand better the impact of adherence”, “shows them why they need to take their pills” and “empowers them on how to solve their own problems”. As one counsellor highlighted: “The use of video makes clients remember to take their drugs on time for it explains about the replication of the virus which leads to drug resistance…So clients have a better understanding of how ART works and what to do to remain healthy. The use of stickers also helps clients to remember taking their drugs on time.” They spoke about the “client-centred” or “patient-driven” quality of the intervention, and how this helped the client to “open up about barriers to adherence”, helped them to “connect with clients” and that clients “seem to respond well to being the driving force”. As one ADC explained, “Basically, the clients could take the lead in decision making and coming up with solutions to their problems, I was only there to guide them. So you see this is what I liked about New Direction. And I know the clients find it very helpful”. Another ADC specifically compared it to standard care and stated why they thought the intervention was better for their clients: “It is different to standard care in that the client controls the sessions. In standard care, the client is not given the chance to think on their own. Basically, it’s like spoon feeding. But with New Direction, it is the client who will be in control and this helps them to empower themselves and think for themselves”. ADCs in both arms of the study liked that sessions were delivered by the same counsellor, which they reported built client-counsellor trust. ADC’s felt this helped lead to “good adherence” because: “There is continuity with the same counsellor and this means the client will have a good rapport with the counsellor, and opens up with everything, which leads to good adherence…You really get to know the client, and so you learn the root cause of poor adherence”. Again the ADCs compared this with standard care and stated this was an improvement: “I liked the fact that the clients really opened-up, and you see it’s because you were doing a follow up. It’s like that with New Direction—when you see a client the first time you don’t just leave them but they will come for more sessions. This is unlike standard care where we just see the client once, and then after that they will see a different person…So it is really good for monitoring the adherence”. ADCs explained that many clients face considerable financial difficulties, and thus providing small research incentives of bus fare and refreshments facilitated a high uptake of care. One ADC explained “The idea of giving clients incentives is very acceptable to clients and reduces the issue of missed appointments. I mean the bus fare motivates the client to come for the sessions. The client will know that whenever I go for the sessions I will get the bus fare and when I am hungry or thirsty I will get refreshments. Many clients at our clinic are failing to come due to financial problems”. The ADCs and the two senior clinic staff felt that the PST-AD intervention should be continued and scaled-up as long as human resources were adequate, as the extra sessions were not part of standard care.

**Table 4:** Key themes from qualitative interview

Theme	Quote
Acceptability and benefits of the intervention (Interviews with intervention arm participants)	“The project should continue educating me on how to take my medications and to deal with my memory loss” “They should help everyone because this is extremely beneficial” “Tendai should consider educating more people on depression and adherence” “I learnt the importance of adherence—if I do not adhere it means that the viral load will rise and in turn will affect my health” “I benefitted a lot from all the sessions I did not understand the difference between viral load and CD4 but through the sessions now I know the difference. I can now manage to solve my problems on my own” “I am now make use of reminders for myself, such as stickers and alarms so that I will be able to take my pills on time and I am now getting condoms from a clinic” “I have learnt that I can do something with the little I have and become someone … I am translating for other people especially in church and I am saving out of it” “With the help of the counsellor, I managed to come up with a solution to my financial problem. I have now started my own business of selling food”
Acceptability and benefits of the intervention (Interviews with ADCs and staff) Client-centred focus Continuity of care Incentives for participants	“The use of video makes clients remember to take their drugs on time for it explains about the replication of the virus which leads to drug resistance…So clients have a better understanding of how ART works and what to do to remain healthy. The use of stickers also helps clients to remember taking their drugs on time” “I think Nzira Itsva should definitively be implemented instead of standard care. We have all learnt a lot, and we have seen that the patients have benefitted” “I think it should definitively be used in all hospitals. Absolutely” “It has been a great benefit to our patients. Particularly the problem-solving component. It addresses some of the psychological and psychosocial factors affecting adherence. The introduction of screening for depression that came with the new intervention also had a positive impact on the way counselling is being conducted, with patients with depression being picked u.” “In Nzira Itsva clients open-up a lot. For example when you ask a patient where they store their medications, they will end up saying there is stigma, which leads them to put their drugs under the bed…they stay with their relatives, which make it difficult. So you then know that these are the major problems… and so together we can then think about how to address these challenge.” “Basically, the clients could take the lead in decision making and coming up with solutions to their problems, I was only there to guide them. So you see this is what I liked about New Direction. And I know the clients find it very helpful” “It is different to standard care in that the client controls the sessions. In standard care, the client is not given the chance to think … it’s like spoon feeding. But with Nzira Itsva, it is the client who will be in control and this helps them to empower themselves and think for themselves” “There is continuity with the same counsellor and this means the client will have a good rapport with the counsellor, and opens up with everything, which leads to good adherence… You really get to know the client, and so you learn the root cause of poor adherence” “I liked the fact that the clients really opened-up, and you see it’s because you were doing a follow up. It’s like that with Nzira Itsva—when you see a client the 1st time you don’t just leave them but they will come for more sessions. This is unlike standard care where we just see the client once, and then after that they will see a different person…So it is really good for monitoring the adherence” “The good thing about these sessions is continuity…that you have a number of sessions and don’t shift counsellors. I think this is very helpful because you will then be able to get a better understanding of the client’s problems. If the client keeps seeing different counsellors, it is not good as none of the counsellors will have a good understanding of the client’s problems” “The idea of giving clients incentives is very acceptable to clients and reduces the issue of missed appointments… the client will know that whenever I go for the sessions I will get the bus fare and when I am hungry or thirsty I will get refreshments” “Many clients at our clinic are failing to come due to financial problems”
Potential intervention contamination (Interviews with EUC counsellors)	“It was wonderful working with the clients who were on the Tendai study. Every time they came to me I knew exactly what to do. I had to concentrate on adherence issues. Because they were on the Tendai study I gave them more attention and time than I usually do with my other clients. That helped a lot because I was able to understand why they had adherence problems and was able to help them which we hardly do with the other clients” “I knew that people in the Tendai Study had depression so I took my time to focus on the problems that were causing them stress and we did stress management” “I gave them more time to talk because of the sensitivity of depression which we do not normally do and being able to air their views helped me understand their problems better and was able to help”

#### Experience of Electronic Adherence Monitoring

Responses suggested that the Wisepill had an effect on participants’ medication behaviour. Common comments included, “it helped me a lot”, “it was very helpful”, “it had a positive effect” and “with the Wisepill I managed to take my pills very well. I was taking them on time”. Indeed, 50% of the EUC participants stated that it aided them in taking their pills on time, while 75% declared that they were more likely to take their medication when using the Wisepill than without it. Several participants in both arms said that the device gave them a sense of “feeling monitored”.

#### Contamination Risks of conducting a Trial in One Clinic

Interviews with the four counsellors who had delivered the EUC arm revealed that their awareness of the study may have impacted significantly on the way in which they treated the EUC participants and conducted the counselling sessions with them. Although the EUC counsellors were asked not to do anything differently other than to provide additional sessions of usual care, it emerged from the interviews that, as they were aware that the participants in the intervention arm of the Tendai study were improving, and had heard of the problem-solving approach “through the cubicle curtains”, they were motivated to help their patients produce similar positive results. For example, one counsellor explained that, because she had some understanding of what the study was about, she “knew exactly what to do” and felt she should “give those patients more attention and time than I usually do with my other clients.” Similarly, another counsellor indicated that he “was aware that any patient coming through Tendai would have depression” and thus “did not concentrate on nutrition and other issues as their problems were mental issues”, while another said she now knows that “if you pay more attention to the patient and give them more time to talk about their problems the outcomes of adherence would improve”. Indeed, all four counsellors stated that they gave the study participants a lot more time and information, in comparison with their other patients. As one counsellor put it, “I was more caring with the Tendai patients”.

Further contamination was revealed through interviewing the ADC who delivered the PST-AD intervention. It emerged she had given two sessions of PST-AD to one EUC arm participant and one session each to 6 EUC arm participants because EUC counsellors referred these clients to her after their EUC sessions due to concerns about the participants’ psychosocial issues.

## Discussion

This is the first study to show the feasibility and acceptability of a model of care to enhance adherence and treat depression in a low-income country in SSA. This model involved task-sharing and stepped care, with trained and supervised adherence counsellors providing six sessions of PST for both adherence and depression, with options embedded to refer to a specialist based on follow-up outcome measures. We have provided evidence of the acceptability of the intervention to both participants and adherence counsellors, and of high fidelity to delivering the intervention at least for initial sessions of therapy. In preparation for a future trial, we have shown feasibility of recruitment, participant randomisation and retention. Although it is relatively common that time-matched controls are not used in effectiveness trials, one limitation was that the study was not a time-matched RCT, having six sessions in the PST-AD arm and three in the EUC arm.

In terms of acceptability, both clients and ADCs appreciated especially the collaborative style of the PST-AD intervention, which put the client “in the driving seat” of their adherence and trained them how to problem-solve for themselves. This is an important finding which fits with evidence from the US on the use of PST for adherence [7] but differs from the standard didactic way of delivering information which is common in HIV clinics in Zimbabwe, and from interventions relying simply on education or reminders [50].

Challenges discovered during the study included the difficulty in relying on routinely collected data to pick up potentially eligible clients at risk of poor adherence to ART, due to weakness in the health records system. For a future trial, recruitment could be enhanced by conducting research measurement to screen for detectable viral load, by outreach screening for patients who miss appointments, and through making use of pharmacy re-fill data as a way to identify patients late for drug pick up [51].

Another important lesson learned for a future trial was about checking fidelity to the adherence component of the follow-up sessions. During the follow-up sessions fidelity to PST for depression was very good, but fidelity to PST for adherence to ART was poor, dropping from 83% in session 1–33% in session 3. For instance, if the individual had financial constraints making it difficult to afford bus fare to collect medication, or family disharmony interfering with achieving a regular medication schedule, the ADC focussed on the underlying problem, often neglecting to review the proximal target of actual adherence behaviour. The ADC also commonly neglected to work on additional barriers to adherence that had been elicited in the first session. This may have been due to the fact that ADCs trained initially as part of our preparatory work were re-located by the hospital matron to critical areas, especially antenatal adherence counselling [11]. This resulted in us having to train a new ADC for the pilot trial, and we may not have sufficiently emphasised the importance of adherence to ART in the follow-up booster sessions. We think that the ADC became engaged with supporting the participants with their depression (for example helping them problem-solve over poverty-related and relationship-related stressors) which she found interesting and rewarding. She felt that these stressors were underlying the client’s problems with adherence and thus she elected to focus on them. However, for the future, it will be important to emphasise that the counsellors should also continue to attend to the proximal target of adherence behaviour. For our future trial, we plan to ensure that this is addressed through training and supervision and with real-time monitoring of fidelity with interim checking.

Another challenge identified through this feasibility trial was contamination. A number of EUC participants were referred into the active intervention and had one or two sessions of PST for depression prior to outcome assessment. Furthermore, the extra sessions freely offered as part of EUC may have helped explain some improvement in the depression and/or adherence levels of the control patients. In addition, adherence counsellors in the EUC arm revealed they had tried to copy what they had heard being delivered in the PST-AD cubicles. This was largely motivated by their wish to learn more, and by pride at developing new skills especially if these can lead to professional certificates. Copying what they could hear being delivered in the intervention arm was facilitated by the lack of privacy. The counselling areas are divided only by curtains. We think our finding is impressive that depression improved significantly more in the PST-AD arm, even in the face of this contamination. For a future trial we will limit contamination risks through having an ADC dedicated to delivering the intervention arm who does not provide routine care, and using a separate private room or cabin. An alternative would be to have some form of placebo control, to allow ADCs in the control arm to gain skills and feel included. Another option would be a cluster design, with clinics being the unit of randomisation.

We demonstrated strong acceptability of the PST-AD intervention through high attendance and through exit interviews. This, combined with evidence from high income countries of the efficacy of combined treatment of depression and adherence using CBT [24–26], provides solid justification for a trial of PST-AD in this low-resource setting where nearly 15% of the population are infected with HIV. PST, although often used as part of CBT, takes its theoretical base from social problem-solving and is an evidence-based treatment for depression that is thought to work through enabling a more positive orientation towards problems and improving coping skills [52, 53].

The very small sample size lead this study to be underpowered, and there may have been missed effects. There were many instances in which there were differences between the PST-AD arm and the EUC arm, e.g. in levels of ART adherence, HIV appointment adherence, and viral suppression, which did not achieve statistical significance, and in future large trial, these differences may emerge as significant. We used electronic monitoring of adherence for the current study, which is considered a robust measure. However, it is expensive and may distort adherence behaviour [54], especially if used as we did only for short periods [55]. An alternative measure of adherence could be Pharmacy Refill Percentage and Appointment Adherence. A future trial could include on-going viral suppression as a primary outcome of interest. Other questions that could be explored through a future, larger trial could be whether ‘stepping up’ mediates any of the improved outcomes, and whether baseline severity moderates outcomes.

Current multidisciplinary guidelines for mental healthcare (e.g. mhGAP) recommend using a stepped care approach to depression, but this has not been investigated previously in PLWH with depression and poor adherence to ART [56]. A strength of our intervention was deploying ADCs to provide Step 1 PST for depression, rather than a nurse or mental health specialist. ADCs are an available cadre who currently deliver adherence support in most low-income countries in SSA [57, 58]. We opted for additional PST by a qualified psychologist for Step 2, but if antidepressants were more generally available in HIV care settings in LMIC, and nurses were trained to prescribe this, this would arguably be more sustainable.

Results from this study inform and justify a future randomised controlled trial of task-shifted PST-AD. If an adequately powered trial mirrors efficacy in enhancing adherence, reducing depression and missed HIV care appointments, and improving HIV viral suppression, this task-shifting model has the potential to be scaled up within existing SSA health care systems. The intervention could have a great impact on the lives of many people living with the dual challenges of HIV and clinical depression in low-resource settings.
